# The effect of pre-heating on monomer elution from bulk-fill resin composites

**DOI:** 10.4317/jced.56989

**Published:** 2020-09-01

**Authors:** Mohammad-Esmaeel Ebrahimi-Chaharom, Leila Safyari, Hossein Safarvand, Elmira Jafari-Navimipour, Parnian Alizadeh-Oskoee, Amir-Ahmad Ajami, Mahdi Abed-Kahnamouei, Mahmoud Bahari

**Affiliations:** 1 Associate Professor, Department of Operative Dentistry, Faculty of Dentistry, Tabriz University of Medical Sciences, Tabriz, Iran; 2 Dental and Periodontal Research Center, Faculty of Dentistry, Tabriz University of Medical Sciences, Tabriz, Iran; 3 Assistant Professor, Department of Operative Dentistry, Faculty of Dentistry, Ardabil University of Medical Sciences, Ardabil, Iran; 4 Professor, Department of Operative Dentistry, Faculty of Dentistry, Tabriz University of Medical Sciences, Tabriz, Iran; 5 Assistant Professor, Department of Operative Dentistry, Faculty of Dentistry, Tabriz University of Medical Sciences, Tabriz, Iran

## Abstract

**Background:**

The present study was aimed to evaluate the effect of pre-heating of bulk -fill resin composites on monomer elution from them.

**Material and Methods:**

Three different types of resin composites were used including Tetric N-Ceram Bulk Fill, X-tra Fill and X-tra Base. 10 cylindrical samples were prepared from each resin composites. Before light curing, 5 samples were pre-heated until reaching 68˚C, then 5 other samples were polymerized at room temperature. After 24 hours, release of UDMA, TEGDMA and BIS-GMA monomers were measured by High-Performance Liquid Chromatography analysis. Data analysis was performed by two-way ANOVA test, Games-Howell and Sidak post hoc tests.

**Results:**

Pre-heating did not have any statistically significant effect on the mean values of UDMA, TEGDMA and Bis-GMA elution (*p*>0.05). The greatest amount of released Bis-GMA and UDMA was obtained from Tetric N-Ceram Bulk-fill composite. The greatest amount of released TEGDMA was obtained from X-tra Fill composite. X-tra Base composite showed the lowest amount of monomer release (*P*<0.001).

**Conclusions:**

Pre-heating did not have any effect on monomer release from bulk-fill resin composites. Moreover, the amount and the type of monomers released from various bulk-fill resin composites were not similar.

** Key words:**Bulk fill composite resin, elution, HPLC, residual monomer, temperature.

## Introduction

Today, the use of resin composite restorations in dentistry has increased due to their esthetic properties and durability. Vidnes -Kopperud *et al.* in their study reported that the dentists’ preference for using composite materials has been increased from 16% to 95% from 1983 to 2009 in Norway (1). Composite materials mainly consist of photo-polymerized methacrylate monomers and fillers. The polymerization rate of composite materials has a great effect on their mechanical strength and durability. Due to incomplete polymerization, unreacted monomers will remain in restoration and may release in the saliva and consequently, cause the diffusion of oral fluids into the restoration. Unreacted monomers and oral liquids both act as a plasticizer which causes a reduction in mechanical resistance and dimensional stability as well as an increase in bacterial growth (2). Moreover, the monomer is also capable to easily penetrate into pulp tissue (3). Experimental studies have shown that monomers have adverse side effects on the oral cells. Due to the production of free oxygen, they damage redox hemostasis and disturb the function of live cells (4). Furthermore, the prior studies showed that monomers like TEGDMA cause an increase in the proliferation of important cariogenic microorganisms, such as *Lactobacillus* and *Streptococcus Sobrinus* (5).

Recently, a new group of resin composites called bulk-fill resin composites has been introduced with the aim of accelerating and facilitating restoration procedure. According to the manufacturer’s claims, they have the ability of photo-polymerization in 4-5 mm of thicknesses, and this is attributed to increased light transmission from these materials (6). Given the tendency of using bulk-fill resin composites in deep cavities near the pulp, some concerns are raised about their biocompatibility on pulp cells, especially if the resin composite is not correctly cured at the cavity floor. Pongprueksa *et al.* in a study compared the degree of conversion and the amount of monomer release in two types of conventional resin composites and one bulk-fill resin composite, and they showed that the degree of conversion in the depth of bulk-fill samples was low and the monomer release was greater in them (3). Therefore, it was found that by using methods for increasing the degree of conversion of resin composites, the amount of monomer release can be reduced.

Pre-heating of the resin composites before placing, would enhance both radical and monomer mobility resulting in higher overall conversion which may improve physical and mechanical features and reduce curing time (7). Furthermore, as a result of the increase in their flowability, adaptation with the prepared cavity walls becomes easier (8). Based on rheological features, bulk-fill resin composites are divided into two categories: low viscosity (flowable) and high viscosity. Pre-heating of high viscosity bulk-fill resin composites may temporarily reduce the viscosity comparable to that of a flowable composite, without sacrificing good mechanical properties (associated with higher filler content) (8). An increase in the polymerization rate and degree of conversion is considered as another advantage of the pre-heating (2). Deb *et al.* in their study showed that increasing the temperature to 60˚C before polymerization in posterior conventional resin composites caused a significant increase in the degree of conversion (2).

Considering that pre-heating influences the degree of conversion (10) and since, the degree of conversion is related to the monomer release (3), the present study was aimed to evaluate the effect of pre-heating on the release of BIS-GMA, TEGDMA and UDMA monomers from three bulk-fill resin composites. Therefore, two null hypotheses were put forth in this study, which were as follows.

First; pre-heating would not have any effect on monomer release from various bulk-fill resin composites. Second, the amount of monomer elution from various bulk-fill resin composites would be similar.

## Material and Methods

**Figure 1:**
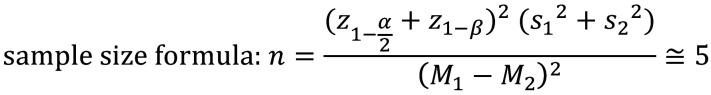
Formula.

For preparation of samples, Teflon cylindrical molds were used with 5 mm diameter and 4 mm height. Three types of bulk-fill resin composites including X-tra Fill (XF), X-tra Base (XB) and Tetric N-Ceram Bulk-fill (TNB) were used (Table 1). Due to the lack of a similar study, pilot study results and sample size formula were used to estimate sample size. (α= 0.05, z1-α/2 =1.96, z1-β= 1.28, M1= 10.29, M2=11.08, S1= 0.27, S2= 0.45), (Fig. 1).

**Table 1:**
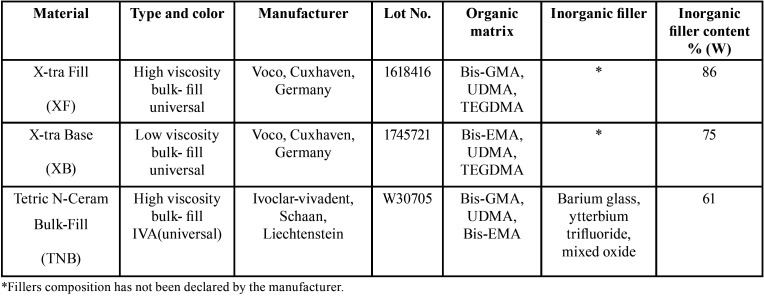
Specifications of used resin composite materials and their abbreviated code.

10 samples were prepared from each resin composite. 5 samples were pre-heated to 68 ˚C before polymerization (7), and 5 other samples were polymerized at the room temperature (25˚C). Therefore, based on the type of resin composite and the pre-heating variable, totally six groups were evaluated in the study (n=5), and total number of prepared composite samples were thirty.

Pre-heating was performed using warm bath equipped with thermostatic control (TELEDYNE HANAU, Buffalo, NY, USA). The material temperature was measured using digital microprobe GBC KDM 350, KON (EL CO SPA, Milano, Italy) (11). The heating time was 5 minutes based on the pilot study.

Teflon molds were completely filled with a single bulk increment of resin composites, and transparent strips were placed on the upper and lower parts of the cylinders to prevent the formation of oxygen-inhibited layer. Then, they were cured using light cure unit (LITEX 695C Cordless LED Curing Light, Dentamerica, USA) with intensity of 1100 MW/cm2 for 20 seconds (3). Before each use of light cure unit, a calibrated radiometer (Bisco, IL, USA) was used to verify the irradiance. For simulation of the clinical conditions, the head of light cure unit was placed in direct contact with one side of the cylinder (12). After polymerization, each sample was immediately immersed in 75 wt% ethanol/water solution as extract fluid and was stored in amber-colored bottles at the room temperature. After 24 hours, 0.5 ml of the extract fluid was taken for High-Performance Liquid Chromatography analysis (HPLC), and the elution was measured for BIS-GMA, TEGDMA and UDMA monomers (13). In order to measure the amount of monomer release, the 600E waters System Controller HPLC device was used (Waters, MA, USA).

**Figure 2:**
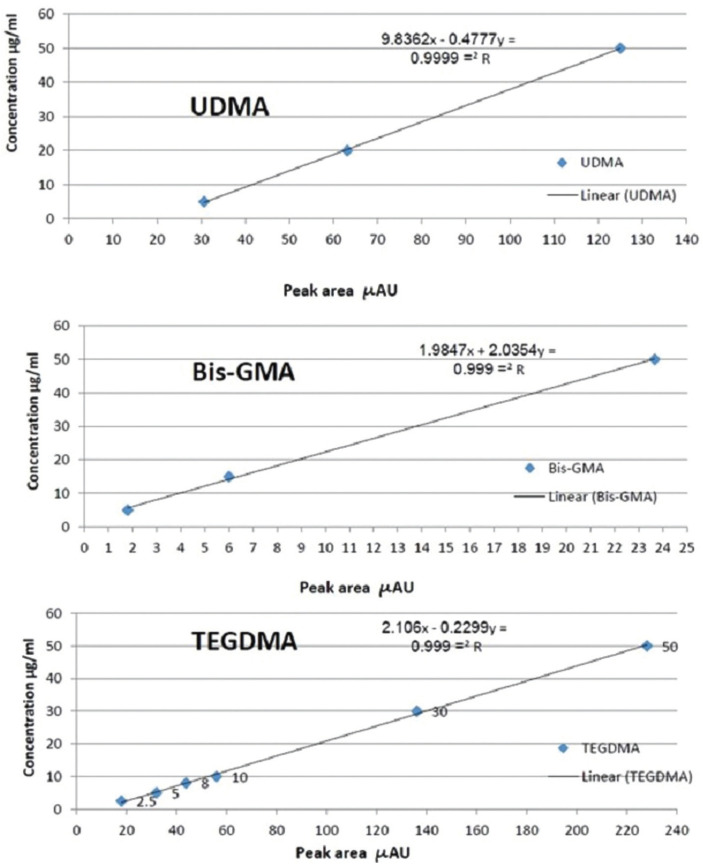
Calibration curves relating to TEGDMA monomer, UDMA monomer and Bis-GMA monomer.

The Perfect target ODS-3 column with 125 mm length and 4 mm diameter and the UV SPD (Spectrophotometric Detector) with a wavelength of 230 nm were used (14). The mobile phase included 70% of acetonitrile and 30% of distilled water, flow rate equal to 0.8 ml/min and 20 microliter injection volume at the room temperature. Various concentrations (from 0.5 to 50 microgram/ml) of the pure samples of each monomer were prepared as reference standards (monomers specifications are provided in Table 2) and were injected to the device. The peak of curves related to each concentration was specified. Then, calibration curves were drawn for each monomer which indicates the linear relation between various concentrations and the area under curve (Fig. 2). R coefficient obtained by linear regression analysis of the calibration curve for all three monomers was equal to 0.999. Based on these equations, the area under curve relating to each sample concentration was obtained, and this obtained concentration was the rate of monomer release in µg/ml. The least quantification limit related to TEGDMA was equal to 1 µg/ml, followed by 1 µg/ml for Bis-GMA, and 2.5 µg/ml for UDMA. Retention time was found to be equal to 2, 3.2, and 2.65 minutes for TEGDMA, Bis-GMA, and UDMA, respectively. In this study, except standard references, all used chemical materials (ethanol and acetonitrile (Merck, Germany) were of liquid chromatographic grade.

**Table 2:**
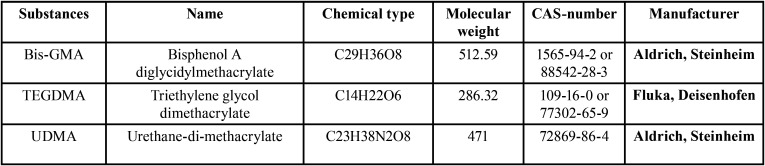
Specifications of the studied monomers.

Statistical analysis:

The Kolmogorov-Smirnov test was used to assess the data normality. In order to evaluate the effect of pre-heating on BIS-GMA, UDMA, TEGDMA monomers release, based on the types of composite and monomers, two-way ANOVA tests were performed separately. Games-Howell and Sidak tests were also used as the post hoc tests. Data were analyzed using SPSS software version 17. The *P* value of 0.05 was considered as statistically significant.

## Results

**Figure 3:**
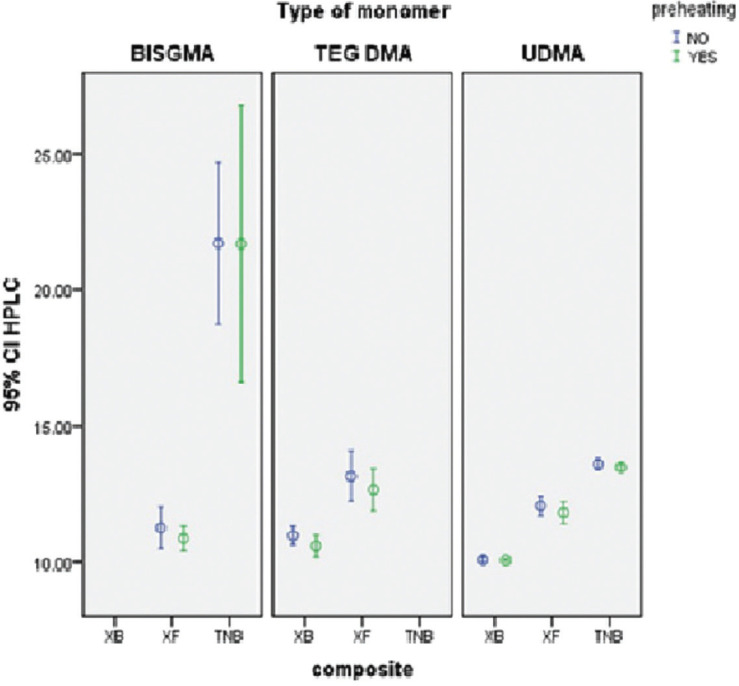
Error-Bar curve of monomers elution based on pre-heating and composite type.

Mean values and standard deviations of monomers release based on pre-heating and type of composites are provided in Table 3 and Figure 3. Bis-GMA monomer was not detecTable from samples prepared from XB composite, and TEGDMA monomer was not delecTable from samples prepared from TNB composite. The results of the Kolmogorov- Smirnov test showed that the distribution of data was normal. Therefore, in order to investigate the study hypotheses, the parametric test was used (*P*>0.05).

**Table 3:**
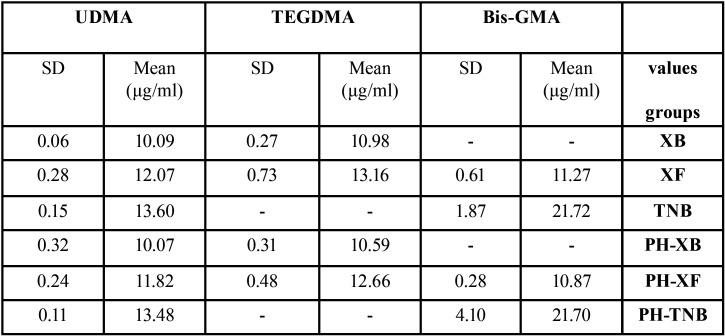
Mean values and standard deviations of various monomers in the studied groups.

The results of the Two-way ANOVA tests performed separately for each monomer showed that the effect of pre-heating on monomer elution was not statistically significant (*p*>0.05), but the type of resin composites had statistically significant effect (*p*<0.001). In addition, there was no relationship between pre-heating and type of resin composites (*P*>0.05). The amount of released Bis-GMA monomer was significantly higher in TNB composite than XF composite (*P*<0.001). The amount of released TEGDMA monomer was significantly higher in XF composite than XB composite (*P*<0.001). The results of Games-Howell test showed that, the elution of UDMA monomer in XB composite was significantly less than that of both XF and TNB composites, and the elution of UDMA monomer in TNB composite was significantly higher than that of XF composite (*P*<0.001).

The results of the Two-way ANOVA tests performed separately for each type of resin composite showed that, the effect of pre-heating on elution of different monomers was not statistically significant (*p*>0.05), but the type and amount of monomer released from the resin composites was significantly different (*p*<0.001). In addition, there was no relationship between pre-heating and type of monomers (*P*>0.05). The elution of TEGDMA monomer was higher in XB composite than that of UDMA monomer (*P*<0.05) and the amount of BISGMA monomer elution was significantly higher than that of UDMA monomer in TNB composite (*P*<0.05). The results of the Sidak test showed that, Bis-GMA monomer elution was significantly less than the two other studied monomers in XF composite (*P*<0.05), and the amount of TEGDMA monomer elution was significantly higher than that of UDMA monomer in XF composite (*P*<0.05).

## Discussion

Several studies have shown that pre-heating causes an increase in the degree of conversion of resin composites (10) and the degree of conversion has been found to be directly related to monomer elution (3). On the other hand, the biocompatibility of the resin composites depends on the quality and quantity of the monomers and other components released from them (15). In the present study the amount of Bis-GMA, TEGDMA, UDMA monomers elution from bulk-fill resin composites including X-tra Base, X-tra Fill, Tetric N -Ceram Bulk-fill was investigated with and without pre-heating.

In the present study, measurement of monomer elution was performed using HPLC method, which is the standard method for investigating monomer release from resin composites (16). In other measurement methods like GC-Mass, monomers with high molecular weight like BISGMA, UDMA are decomposed in gas chromatography and only the products obtained from their decomposition will be detecTable. Compared to GC-Mass method, HPLC showed better accuracy and control (17). In this method, various factors like chemical features of the solvent, the degree of conversion of resin composite and chemical nature of released components may influence monomer elution (18). Similar to the study by Lempel *et al.*, in this study ethanol 75% was used as an extraction medium for unreacted monomers release. Due to hydrophobic nature of the monomers, their release in ethanol is more than aqueous environment and as a result, the test duration would become shorter. Though it is believed that aqueous environment is better for simulation of oral conditions, it is difficult to simulate variable parameters such as PH, temperature and enzyme activity (19).

The results of the current study showed that, pre-heating of the used bulk-fill composites caused a decrease in the amount of monomer release from them, but this effect was not statistically significant. So, the first study hypothesis was confirmed. Previous studies have reported that pre-heating of resin composites before curing could increase polymerization rate and degree of conversion (10) through temporary reduction of viscosity (9). Pongprueksa *et al.* in a study also reported that increased degree of conversion resulted in a decrease in the amount of monomer release (3). While, in the study by Almeida *et al.*, no significant effect was reported regarding the administration of pre-heating on degree of conversion (20). Additionally, it was found that, the method effective on the degree of conversion does not necessarily have a significant effect on monomers release. In the study by Manojlovic *et al.*, various light sources (halogen and LED) were used for light curing of Nano hybrid, conventional micro hybrid resin composites, and ormocer. Although various light sources may influence the degree of conversion but did not cause a significant difference in monomers release (16). So, it is believed that, monomer release has a very complicated mechanism and it may be influenced by other factors like chemical composition of the resin matrix and monomer position in the structure (19).

As another considerable finding, the present study showed that, various resin composites released different amount and types of monomers. According to this result, the second hypothesis was rejected, and this result was consistent with the results of other studies (19-21). Lempel *et al.* reported different amounts of released monomers from bulk-fill resin composites (19). The results of the present study showed that, the release of Bis-GMA and UDMA monomers from TNB composite was more than those of the other two resin composites. Possibly, higher percentage of filler -to -matrix ratio resulted in a decrease in the composite solubility (22). Several studies showed that in resin composites with more filler content, the solvent absorption is lower than resin composites containing less filler (22,23). Moreover, less absorption of solvent may cause fewer release of components (19). According to the manufacturer’s information, the filler content (expressed as weight %) of bulk-fill composites used were as follow: 61%, 86%, 75% for TNB, XF and XB, respectively. So, the lower amount of monomer release from XF composite may be related to higher filler load of this composite compared to other composites. On the other hand, TEGDMA monomer in composites structure has a synergic effect on degree of conversion (19). According to the manufacturer information, there is no TEGDMA monomer in the chemical structure of TNB composite, as confirmed by the results of the present study indicating TEGDMA monomer was not detected from that. This molecule exists in the structure of XB and XF composites and may cause an increase in the polymerization rate and reduction in the monomer elution compared to TNB composite. Moreover, TNB composite contains pre-polymerized fillers. Residual unreacted double carbon-carbon bands in these fillers may result in an increase in the amount of leachable monomers (16). The findings of the present study revealed that the released amount of TEGDMA, UDMA monomers from XB composite were less than those of XF composite. Numerous studies have reported that, the degree of conversion in XB composite is higher than that of XF composite (24,25), which may justify the less monomer release from XB composite. Additionally, according to the manufacturer information, Bis-GMA does not exist in XB composite structure, also the results of this study confirmed that, this monomer was not released from XB composite. Łagocka *et al.* in a study investigated the amount of monomer release from bulk-fill resin composites, and they showed that, any of the studied monomers such as Bis-GMA was not released from XB composite (21). Bis-GMA molecule is a monomer with high molecular weight, high capacity of hydrogen band and low molecular movement (26). In the structure of XB composite, Bis-GMA molecule has been replaced with Bis-EMA molecule, which is a monomer with low viscosity and greater reactivity. It should be considered that XB composite is a flowable bulk-fill resin composite. The low viscosity of this composite has a great effect on the migration of free radicals in it. These factors may lead to improvement of the degree of conversion related to this composite, and may be effective in less release of monomer from it. Contrary to this result, Lempel *et al.*, found that. Bis-GMA molecule was released in low values from XB composite which has been attributed to its matrix impurity (19).

The results of the present study showed that, the amount of released TEGDMA monomer was found to be more in XF and XB resin composites than other monomers. Also, in the study by Lempel *et al.* (19), the greatest amount of released monomer from studied composites has been reported for TEGDMA monomer. This monomer has a low molecular weight and also has an ethylene oxide group in its structure. This ethylene oxide group may cause an increase in the monomer reactivity, its mobility and release in the solvent (21). Though, Alshali *et al.* (27), in a study showed that, TEGDMA, UDMA monomers were not released from XB composite in an aqueous environment, but in this study, the release of monomers from XB composite was shown to be detecTable in the ethanol environment.

Due to the known toxicity of Bis-GMA monomer, the release of this type of monomer has been investigated in most studies (3,13,19,21,27). The results of the present study showed that, the release of Bis-GMA monomer from XF resin composite was less than that of UDMA, TEGDMA monomers. Other studies showed similar results and reported lower release of Bis-GMA than other monomers from bulk-fill resin composite (21,27). Bis-GMA molecule has high viscosity and molecular weight and low mobility. These features may lead to less release of this type of monomer. A combination of relatively high molecular weight along with a high concentration of double bands and low viscosity has been found in UDMA monomer (19). These factors may lead to more release of UDMA monomer compared to Bis-GMA monomer.

The amount of UDMA monomer release was less in TNB resin composite than Bis-GMA monomer. The amount of monomer release from a resin composite not only depends on the amount of its unreacted monomer and monomer nature, but also may be related to the chemical structure of resin matrix and monomer position in the polymer network. In polymerized resin, monomers that are stuck in micro porosities are more susceptible to elution, and heterogeneous materials have higher volume of micro porosities (21).

In the current study, the release of limited monomers was investigated and measurements were done only after 24 hours. Investigation of other monomers and in longer period of time may provide more interesting results. Also, the comparison of different extraction mediums and the effect of various curing methods on monomer release from resin composites are suggested for future studies.

The results of the present study showed that, pre-heating of bulk-fill resin composites up to 68˚C did not influence on release of BIS-GMA, TEGDMA, and UDMA monomers. Moreover, the amount and type of released monomers from different bulk-fill resin composites with various viscosities were not similar.
